# *unc-119* mutants have an increased fungal spore** adhesion that is not rescued by *Cb-unc-119*

**DOI:** 10.17912/micropub.biology.000344

**Published:** 2021-01-05

**Authors:** Shizue Omi, Nathalie Pujol

**Affiliations:** 1 Aix Marseille Univ, INSERM, CNRS, CIML, Turing Centre for Living Systems, Marseille, France

## Abstract

If the cuticle acts as a protective barrier against environmental insults, several pathogens have developed strategies that use it as a way to infect *C. elegans*. The fungus *Drechmeria coniospora* produces spores that attach to the cuticle, before hyphae invade the body. Mutants with an altered surface coat, the outermost layer of the cuticle, including *bus-2*, *bus-4*, *bus-12* and *bus-17* show increased adhesion of fungal spores (Rouger et al, 2014; Zugasti et al, 2016). We unexpectedly found that *D. coniospora* spores attach unusually densely around the mouth of *unc-119* mutants. Interestingly, this phenotype is not rescued by the *C. briggsae* *unc-119* construct that is conventionally used to rescue neuronal *unc-119* phenotypes.

**Figure 1. unc-119 mutants are highly susceptible to infection by the fungus Drechmeria coniospora:**
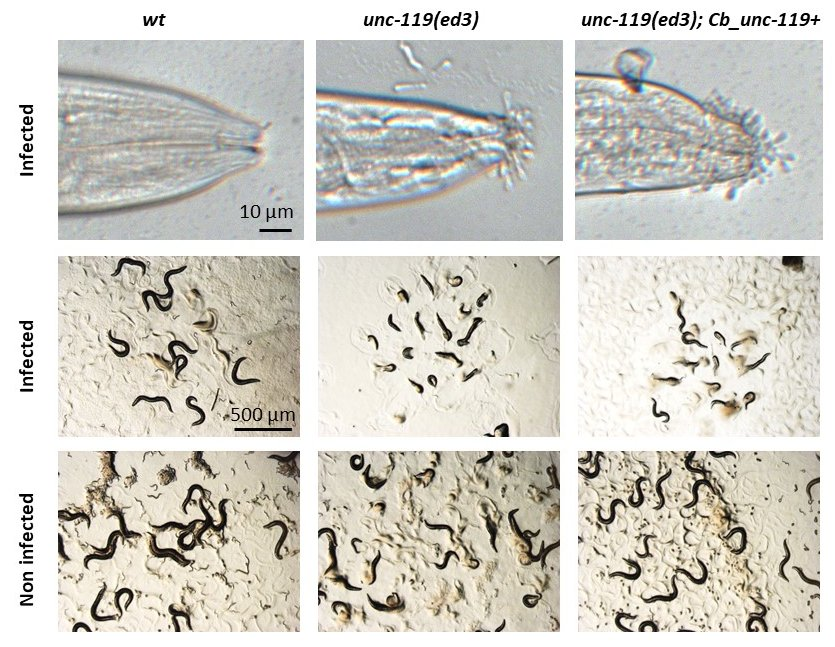
After 24 h of infection by *D. coniospora*, *C. elegans* young adult worms were observed for the adhesion of spores at the mouth (upper row) or their overall morphology (middle row). Non-infected worms are shown for comparison (lower row). *unc-119* mutant worms carrying a wild-type copy of *Cb-unc-119* (*unc-119*(+), right column) or not (middle column) had an increased number of spores adhering to the mouth and an increased susceptibility to infection (reflected by a reduction in size), compared to wild-type worms (left column, *wt*).

## Description

As part of our investigations of the interaction between *C. elegans* and *D. coniospora*, we made use of MosSCI strains, constructed in an *unc-119(ed3)* background (Frøkjær-Jensen *et al.*, 2008; Maduro, 2015). We noticed that a variety of these strains exhibited a greatly increased susceptibility to infection. Upon further examination, we determined that this was due to an increase in the adhesion of fungal spores, most prominently at the tip of the head (Figure 1). The phenotype was not observed in a strain carrying a wild-type *C. elegans* *unc-119* rescuing construct in an *unc-119(e2498)* background. But the increased spore adhesion was visible in both the *unc-119(ed3)* and the *unc-119(tm4063)* background even in the presence of the standard *C. briggsae unc-119* rescuing construct. The phenotype was absent from these same transgenic strains in which *unc-119(ed3)* was eliminated by out-crossing (Table 1 below). *unc-119* function has been extensively analysed in the nervous system. Notably, some expression in the epidermis was reported recently (Lear *et al.*, 2018). While we have not determined the precise cause, since spore adhesion is a major determinant of infection progression (Zugasti *et al.*, 2016), such effects need to be taken into account when interpreting experiments involving any strain that has an *unc-119* allele in it, which has been often employed as selectable marker for transgenesis.

**Table:** 

strain	genotype	spore adhesion
N2	*wt*	normal
EG6699	*unc-119(ed3) III; ttTi5605 II*	increased
IG1604	*unc-119(ed3) III; frSi6[col-154p::CEBP-1::GFP::3’cebp-1, Cb-unc-119(+) ttTi5605] II*	increased
IG1633	*+; frSi6[col-154p::CEBP-1::GFP::3’cebp-1, Cb-unc-119(+) ttTi5605] II*	normal
IG1622	*unc-119(ed3) III; frSi9[pNP151(col-62p::Lifeact::mKate_3’c-nmy), Cb-unc-119(+) ttTi5605] II*	increased
IG1623	*+; frSi9[pNP151(col-62p::Lifeact::mKate_3’c-nmy), Cb-unc-119(+) ttTi5605] II*	normal
IG1629	*unc-119(ed3) III; frSi10[pNP150(F40H7.12p::GFP), Cb-unc-119(+) ttTi5605] II*	increased
AX6672	*unc-119(ed3) III; npr-1(ad609); ilcr-1(tm5866); [ilcr-1p::loxp::ILCR-1::let-858 3’UTR , Cb-unc-119(+) ttTi5605] II*	increased
OP533	*unc-119(tm4063) III; wgIs533[CEH-18::TY1::EGFP::3xFLAG, Cb-unc-119(+)]*	increased
JR667	*unc-119(e2498::Tc1) III; wIs51[SCMp::GFP, Ce-unc-119(+)] V*	normal

## Methods

Eggs, prepared by the standard bleach method, were allowed to hatch in 50 mM NaCl in the absence of food at 25°C overnight. Synchronized L1 larvae were transferred to NGM agar plates spread with *E. coli* OP50 and cultured at 25°C until the L4 stage (40 h) before being exposed to fungal spores as previously described (Pujol *et al.*, 2001). Images were taken of worms mounted on a 2% agarose pad on a glass slide anesthetized with 0.25 mM levamisole, using a Zeiss AxioCam HR digital colour camera and AxioVision Rel. 4.6 software (Carl Zeiss AG).

## Reagents

N2, EG6699 *unc-119(ed3); ttTi5605 II,* JR667 *unc-119(e2498::Tc1) III; wIs51[SCMp::GFP, Ce-unc-119(+)] V* and OP533 *unc-119(tm4063) III; wgIs533[CEH-18::TY1::EGFP::3xFLAG, Cb-unc-119(+)]* strains were provided by the CGC (*Caenorhabditis* Genetics Center), which is funded by NIH Office of Research Infrastructure Programs (P40 OD010440). In addition, the following strains were tested for spore adhesion: IG1604 *unc-119(ed3); frSi6[col-154p::CEBP-1::GFP::3’cebp-1, Cb-unc-119(+) ttTi5605] II* (Kim *et al.*, 2016), IG1622 *unc-119(ed3); frSi9[pNP151(col-62p::Lifeact::mKate_3’c-nmy), Cb-unc-119(+) ttTi5605]* *II*, IG1623 *frSi9[pNP151(col-62p::Lifeact::mKate_3’c-nmy), Cb-unc-119(+) ttTi5605] II* (Taffoni *et al.*, 2020), AX6672 *unc-119(ed3); npr-1(ad609); ilcr-1(tm5866); [ilcr-1p::loxp::ILCR-1::let-858 3’UTR, Cb-unc-119(+) ttTi5605] II* (Chen *et al.*, 2017), IG1633 *frSi6[pNP145(col-154p::CEBP-1::GFP::3’cebp-1), Cb-unc-119(+) ttTi5605] II* and IG1629 *unc-119(ed3); frSi10[pNP150(F40H7.12p::GFP), Cb-unc-119(+) ttTi5605] II,* this study. Both pNP145 and pNP150 were derived from pCFJ151 – ttTi5605_MCS, that was a gift from Erik Jorgensen (Addgene plasmid # 19330 ; http://n2t.net/addgene:19330 ; RRID:Addgene_19330) (Frøkjær-Jensen *et al.*, 2008).
